# Evaluation of a Novel Autostereoscopic Three-Dimensional System for Binocular Visual Function: A Comparative Validation Study

**DOI:** 10.3390/jcm15103670

**Published:** 2026-05-10

**Authors:** Danjie Han, Yangyi Huang, Ziqing Ding, Jingrong Liu, Jifang Wang, Xingtao Zhou, Wen Wen, Yang Shen

**Affiliations:** 1 School of Health Science and Engineering, University of Shanghai for Science and Technology, Shanghai 200093, China; 232462708@st.usst.edu.cn; 2 Eye Institute and Department of Ophthalmology, Eye & ENT Hospital, Fudan University, Shanghai 200031, China; docthuangyangyi@163.com (Y.H.); 20301050243@fudan.edu.cn (Z.D.); emcris@sina.com (J.L.); wangxuan7907@163.com (J.W.); doctzhouxingtao@163.com (X.Z.); 3 NHC Key Laboratory of Myopia (Fudan University), Key Laboratory of Myopia, Chinese Academy of Medical Sciences, Shanghai 200031, China; 4 Shanghai Research Center of Ophthalmology and Optometry, Shanghai 200031, China; 5 Shanghai Engineering Research Center of Laser and Autostereoscopic 3D for Vision Care, Shanghai 200031, China

**Keywords:** autostereoscopic 3D technology, anaglyphic 3D technology, Titmus stereopsis test, binocular visual function, agreement assessment

## Abstract

**Background/Objectives**: Autostereoscopic three-dimensional (3D) technology represents a promising tool for ophthalmic examinations. However, prior studies have largely focused on stereopsis alone in cooperative adults, leaving comprehensive validation across multiple binocular domains and diverse patient groups unexplored. This study introduces the head-to-head evaluation of a novel integrated, glasses-free autostereoscopic 3D system. The system could simultaneously assess four essential binocular functions (simultaneous perception, fusion, stereoacuity, and stereopsis), with a single automated workflow featuring real-time eye tracking. This study aimed to assess the comparative performance of the novel autostereoscopic 3D display in evaluating binocular visual function, as a preliminary potential screening tool benchmarked against conventional screening tests. **Methods**: A total of 232 participants (age: 3–60 years; male/female: 125/107) were recruited. All participants sequentially underwent binocular visual function assessments using autostereoscopic 3D technology, anaglyphic 3D technology (red–green glasses), and the Titmus stereopsis test. Pearson correlation analysis, Phi coefficient, Cohen’s Kappa and Bland–Altman plots were conducted to assess the consistency. Receiver operating characteristic curve analysis was employed to evaluate the screening comparative performance of autostereoscopic 3D technology, using the anaglyphic 3D technology and the Titmus stereopsis test as comparative reference standards. **Results**: Relative to anaglyphic 3D technology, agreement for simultaneous perception (φ = 0.527, κ = 0.425) and fusion (φ = 0.520, κ = 0.503) was moderate (all *p* < 0.001). Compared to the Titmus stereopsis test, agreement for stereoacuity levels (r = 0.779, κ = 0.659) and for stereopsis detection (φ = 0.624, κ = 0.619) was substantial (all *p* < 0.001). The areas under the curve for identifying abnormal binocular visual function using autostereoscopic 3D technology were 0.769 for overall binocular vision function, 0.747 for simultaneous perception, 0.730 for fusion, 0.846 for stereoacuity, and 0.810 for stereopsis (all *p* < 0.001). Sensitivities and specificities ranged from 52.8 to 81.7% and 77.9–93.1%, respectively. **Conclusions**: Autostereoscopic 3D technology demonstrated significant correlation and moderate agreement with traditional methods in assessing binocular visual function, particularly for stereopsis assessment.

## 1. Introduction

The three fundamental components of binocular visual function—simultaneous perception, fusion and stereopsis—play crucial roles in perceiving three-dimensional space [1,2]. Abnormal binocular visual function can lead to diplopia, visual fatigue, and impaired stereopsis, which may indicate conditions such as strabismus, amblyopia, anisometropia, or neurological disorders [3,4]. These conditions impose a substantial public health burden. The global pooled prevalence of amblyopia in children is estimated at 1.36% [5], while the overall prevalence of strabismus is approximately 5.56% in eastern China [6]. The prevalence of strabismus in Scotland is approximately 2%, whilst the prevalence of binocular vision abnormalities ranges from 3.02% to 4.89% [7]. Moreover, stereopsis deficits are present in up to 15–29% of children with refractive errors [8]. Studies show that fusion and stereopsis deficits in children and adolescents often precede myopia onset, serving as early warning signs for refractive errors [9,10]. Currently, clinical methods for assessing binocular visual function include the Worth 4-dot test (red–green dissociation), polarized dissociation, the Titmus stereogram, the TNO random-dot stereogram, and the synoptophore [11,12]. These techniques rely on red–green and polarized glasses to achieve binocular dissociation, thereby presenting simulated 3D vision [13]. These techniques are associated with alterations in color perception and brightness. Additionally, the fixed patterns used in the Worth 4-dot test and Titmus test may induce learning effects during follow-up examinations. And they typically require one-on-one assessment by trained examiners [14,15]. Conventional methods lack automated quality control, allowing unreliable results from poor fixation or attention lapses to remain undetected [16,17].

The novel autostereoscopic three-dimensional (3D) technology system evaluated in this study uniquely addresses these limitations through integrated automation and multi-domain assessment. By combining real-time eye tracking (ensuring valid gaze position), a glasses-free display (eliminating hygiene and logistical barriers), and standardized computerized protocols (reducing examiner dependence), this system enables efficient, hygienic screening suitable for high-throughput environments. Crucially, it is an all-in-one platform that integrates four essential binocular domains—simultaneous perception, fusion, quantitative stereoacuity, and dynamic stereopsis—within a single automated workflow, potentially providing comprehensive binocular visual function screening without the need for specialized personnel or equipment. Previous studies have mainly focused on adults who were highly cooperative, healthy, or myopic, with limited validation to stereopsis [9,18]. Recently, autostereoscopic 3D tablets have been adapted for clinical stereopsis testing (e.g., the ASTEROID test), demonstrating the feasibility of glasses-free displays in quantifying stereoacuity without monocular cue artifacts [19]. Meanwhile, eye-tracking technologies integrated with automated displays are increasingly being applied to pediatric binocular vision assessment, offering objective gaze-vector analysis for detecting subtle oculomotor deviations [20]. Furthermore, smartphone-based deep learning systems have shown promise for early detection of visual impairment in young children, underscoring a broader paradigm shift toward automated, scalable ophthalmic screening [21]. However, comprehensive feasibility studies on binocular visual function (including simultaneous perception, fusion, and stereopsis) in diverse patient groups, such as children and those with strabismus or amblyopia, remain largely unexplored.

This study aimed to comprehensively evaluate the capability of this novel integrated autostereoscopic 3D technology for multi-domain binocular visual function assessment, ranging from normal to severely impaired. This was done by comparing its correlation and consistency with conventional tests, as well as evaluating its comparative performance in screening for abnormal binocular visual function. The study assessed the preliminary potential of autostereoscopic 3D technology for large-scale binocular visual function screening in school or community settings.

## 2. Materials and Methods

### 2.1. Study Design and Participants

This was a single-center, prospective study. A total of 232 participants aged 3–60 years were recruited from the Shanghai Eye and ENT Hospital of Fudan University from December 2023 to January 2024. This study was designed as a preliminary validation of a novel autostereoscopic 3D system within a fixed recruitment window. The target sample size was informed by sample sizes reported in similar validation studies of autostereoscopic 3D technology for stereopsis assessment, which have typically ranged from 74 to 135 participants [18,22]. The final sample of 232 participants represented the maximum feasible enrollment given clinical throughput and participant availability during this period. Post hoc power analysis confirmed that this sample size was sufficient to detect moderate correlations and AUC values with adequate statistical power (>90%). The study received ethical approval from the Ethics Committee of the Eye and ENT Hospital of Fudan University (No. 2023069) and was conducted in accordance with the principles of the Helsinki Declaration. Informed consent was obtained from all participants.

The inclusion criteria for this study were as follows: (1) age 3–60 years; (2) best-corrected visual acuity (BCVA) ≤ 0 logMAR; (3) cylindrical power < 4.00 D; (4) absence of systemic diseases. The exclusion criteria included: (1) unstable refractive error (change ≥ 0.50 D in spherical or cylindrical power within the past 6–12 months, or ≥0.75 D difference between two consecutive cycloplegic refractions); (2) acute ocular inflammation, such as infectious conjunctivitis, uveitis, keratitis, or blepharitis; (3) presence of psychiatric disorders.

All participants underwent ophthalmic examinations: slit-lamp examination, BCVA measurement, subjective refraction without cycloplegia, and binocular visual function testing. The binocular function tests were carried out in a fixed order: autostereoscopic 3D technology, anaglyphic 3D technology, and Titmus stereopsis test. This fixed sequence was implemented both due to logistical constraints and to ensure consistency across all examinations conducted by the same experienced examiner (JRL) in the same room. Moreover, the sequence was intentionally designed to prioritize the novel autostereoscopic 3D system, ensuring that any learning effects, neural adaptation, or practice gains would benefit the subsequent reference standard tests rather than the system under evaluation [23]. It should be noted that this fixed sequence may lead to a fatigue effect. While it protects the novel system from artificial inflation by learning effects, visual fatigue from repeated testing may conversely degrade performance on subsequent reference tests, potentially biasing comparative metrics in either direction.

### 2.2. Examination Parameters and Equipment

#### 2.2.1. Autostereoscopic 3D Technology

The autostereoscopic 3D technology was used with a glasses-free binocular visual function assessment device featuring an ultra-high-definition display (Model H090L016, Version 1.0, EVIS Co., Inc., Shanghai, China). The system presented dynamic disparities with continuously variable stereoacuity levels (arcsec). The updated unit integrates a depth camera with a 15.6-inch ultra-high-definition panel (3840 × 2160 pixels; 400 cd/m^2^; contrast ratio 1200:1). The device employed laminated lenticular-grating technology to deliver independent images to each eye.

The device detected eye orientation using intelligent 3D eye detection and tracking technology based on deep learning, and incorporated an intelligent 3D image processor. Eye tracking accurately detected when participants were positioned too close for valid interpretations. Utilizing a full-fit column mirror grating, the device supported color marker tracking and seamlessly toggled between 2D and 3D modes, in addition to allowing manual mode switching. The device permitted a viewing distance of 35–120 cm, a horizontal range of motion of 43.6° and a vertical range of motion of 39.6°.

**Figure 1:**
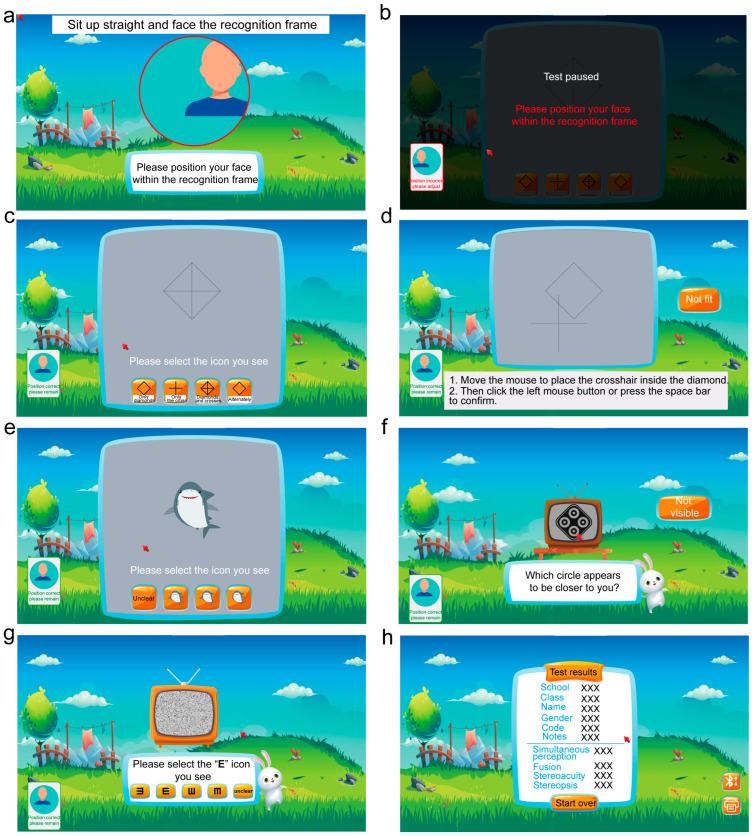
Procedures of the binocular visual function assessment using the autostereoscopic 3D system. (**a**) Eye tracking. (**b**) Test paused due to unstable gaze. (**c**) Simultaneous perception. (**d**) Simultaneous perception offset. (**e**) Fusion. (**f**) Stereoacuity. (**g**) Stereopsis. (**h**) Output result.

The testing procedure is illustrated in Figure 1. The participant’s face was aligned with the recognition frame (Figure 1a). If face recognition failed, the system automatically paused detection (Figure 1b). Participants sat 50 cm from the screen and responded by moving and clicking the mouse. Assessments were conducted sequentially for simultaneous perception, strabismic deviation, fusion ability, stereoacuity, and stereoscopic vision. For simultaneous perception, participants with normal simultaneous perception perceived both the diamond and cross at once. If participants saw only one shape or alternating patterns, this indicated abnormal simultaneous perception (Figure 1c). For strabismus deviation detection, participants moved the mouse to position the cross within the diamond, then confirmed by pressing the left mouse button or spacebar (Figure 1d). During the fusion test, a complete shark was perceived if fusion was intact; missing either arm signified impaired fusion (Figure 1e). Stereopsis testing employed nine sets of four-circle contour stereograms. Participants clicked the left mouse button on the protruding black circles. Participants with severely impaired stereopsis would fail to perceive the raised black circles (Figure 1f). The stereopsis employed random-dot stereograms (Figure 1g). Participants selected the corresponding answer based on the “E”-shaped targets they perceived; if no ‘E’ was visible, they choose “Unclear.” Upon completing all tests, results were presented (Figure 1h).

When recording results, simultaneous perception was classified as “normal”, or “left/right eye suppression”, or “alternating suppression”. Fusion was recorded as “normal” or “abnormal”. Stereoacuity was recorded as 40, 50, 60, 80, 100, 140, 200, 400, or 800 arcsec. Stereopsis was classified as “normal” or “abnormal”.

#### 2.2.2. Anaglyphic 3D Technology

**Figure 2:**
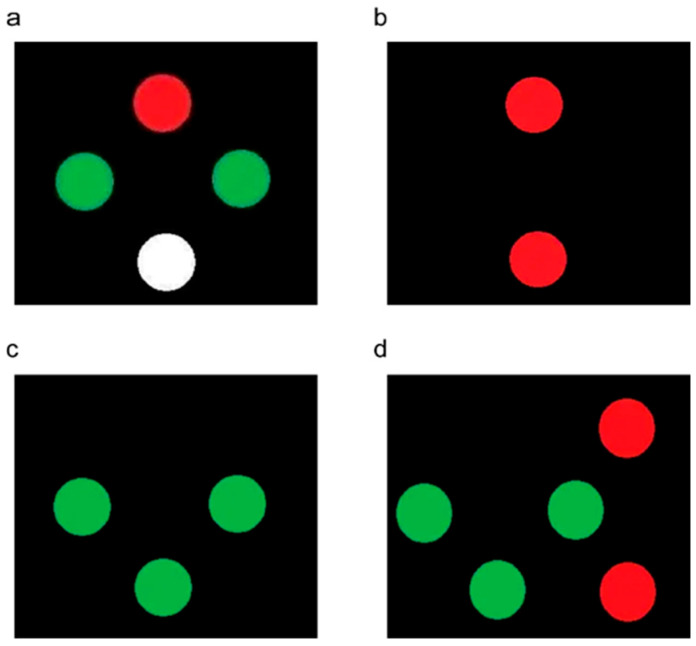
Worth 4-dot test results (anaglyphic 3D technology). (**a**) Normal simultaneous perception and fusion. (**b**) Normal simultaneous perception with abnormal fusion. (**c**) Suppression of the left eye. (**d**) Suppression of the right eye. Participants wore red-green glasses. Red dots represent images perceived by the right eye (through the red filter), green dots represent images perceived by the left eye (through the green filter), and the white dot represents binocular fusion of red and green light.

Simultaneous perception and fusion were assessed using anaglyphic 3D technology (red–green glasses). Participants wore red–green glasses while the examiner employed the Worth 4-dot test to evaluate varying degrees of sensory fusion. The specific procedure was the same as previously reported [24]. Participants with normal sensory fusion saw four images (Figure 2a). If only two images were seen (Figure 2b), it indicated suppression of the left eye, while if only three were seen, this indicated suppression of the right eye (Figure 2c). Participants with normal simultaneous perception but abnormal fusion saw five images (Figure 2d). Notably, this test was prone to false fusion and suppression artifacts [24].

#### 2.2.3. Titmus Stereopsis Test

The fly icon and nine sets of four-circle diagrams (Chicago, USA) were used for quantitative assessment in the Titmus stereopsis test. Each participant wore polarized glasses to perform the stereopsis test and was instructed to follow the testing sequence from the fly to the circle diagrams, with the specific testing process identical to previous reports [25]. First, the participant was shown the fly icon. When viewed monocularly with either eye, the fly had only one layer of wings. When the participant possessed near stereopsis, the participant saw a “three-dimensional fly” with two layers of wings in a clear pattern. If the participant could see the “three-dimensional fly”, nine levels of stereo circles (stereoacuity) were quantified and the results were recorded. Notably, the Titmus stereopsis test was affected by monocular cues. The circle test patterns may be partially visible monocularly, potentially allowing participants with limited or no stereopsis to respond correctly [25]. The exclusion of synoptophore and TNO tests was primarily due to resource constraints and limitations in participant cooperation time (particularly in young children). Furthermore, this study focused on comparing the novel technology against currently used clinical screening methods rather than comprehensive diagnostic standards.

### 2.3. Statistical Analysis

SPSS 26.0 software was used for analysis. Continuous data were expressed as mean ± standard deviation (mean ± SD). To eliminate potential bias from different parallax gradients and enhance comparability, stereoacuity results were recorded as one of four levels, including ≤100 arcsec (level 1), 101–200 arcsec (level 2), 201–400 arcsec (level 3) and ≥400 arcsec (level 4). The Phi coefficient was used to assess the correlation of categorical nominal data (simultaneous perception categories, stereopsis normal/abnormal). The correlation between the Titmus stereopsis test and autostereoscopic 3D measured ordinal stereoacuity levels was analyzed by Pearson correlation analysis. Cohen’s Kappa test was used to assess agreement beyond chance with established interpretation criteria. Bland–Altman plots were employed to compare the consistencies. Wilcoxon’s signed-ranks test was conducted to compare differences in simultaneous perception and stereoacuity levels. McNemar tests were used to compare the level of fusion and stereopsis. ROC analysis was performed to assess the screening comparative performance of the autostereoscopic 3D technology, using the anaglyphic 3D technology (for simultaneous perception and fusion) and Titmus stereopsis test (for stereoacuity and stereopsis) as comparative reference standards (rather than the gold standards for binocular visual function). For simultaneous perception ROC analysis, “left eye suppression,” “right eye suppression,” and “alternating suppression” were recorded as abnormal. For stereopsis ROC analysis, values > 100 arcsec were recorded as abnormal. This categorization was based on clinical relevance, where stereoacuity of ≤ 100 arcsec is generally considered adequate for most daily activities, while values > 100 arcsec indicate clinically significant impairment requiring further evaluation [26]. For overall binocular vision ROC analysis, any abnormality in one of the three visual functions (simultaneous perception, fusion, stereopsis) was recorded as abnormal. *p* < 0.05 was considered statistically significant.

## 3. Results

### 3.1. Descriptive Analyses

The demographic data of the participants is shown in Table 1. The participants’ distribution was male:female = 125:107; adults:children = 37:195; strabismus:post-strabismus correction:refractive error = 137:76:19. Ages ranged from 3 to 60 years, with a mean of 11.4 ± 8.7 years. The binocular difference in spherical equivalent (SE) was +0.03 ± 1.40 D. The cohort was predominantly pediatric (84.0%) and enriched for strabismus (59.1%) and postoperative strabismus (32.8%), with only 8.2% representing participants with uncomplicated refractive error.

### 3.2. Correlation Outcomes

Autostereoscopic 3D technology classified 70% and 79% of participants as having normal simultaneous perception and fusion, respectively, compared with 61% and 69% for anaglyphic 3D technology. Table 2 summarizes the agreement and difference analyses. Autostereoscopic 3D technology showed a significant correlation and moderate consistency with anaglyphic 3D technology for both simultaneous perception (φ = 0.527, κ = 0.425, *p* < 0.001) and fusion (φ = 0.520, κ = 0.503, *p* < 0.001). No significant difference was observed for simultaneous perception (Z = −1.863, *p* = 0.062). By contrast, fusion differed significantly between the two methods (χ^2^ = 10.756, *p* = 0.001).

**Figure 3:**
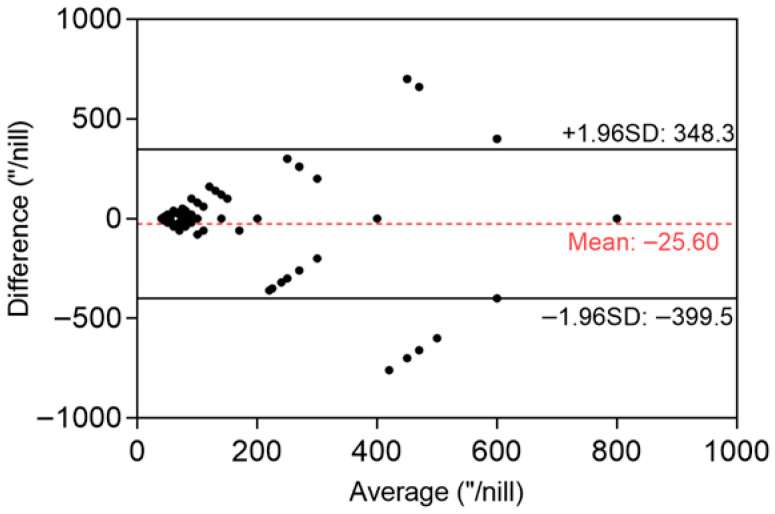
Bland–Altman plot comparing stereoacuity measured with the Titmus stereopsis test and the autostereoscopic 3D method (*n* = 232). The red dashed line represents the mean difference (−25.60 arcsec), and the black solid lines indicate the 95% limits of agreement (−399.5 to +384.3 arcsec).

For stereoacuity levels 1–4, autostereoscopic 3D technology classified 30%, 0%, 6%, and 64% of participants, respectively, versus 25%, 0%, 12% and 64% with the Titmus test. Meanwhile, the percentages of normal stereopsis were 57% and 55%. Table 2 presents the consistency and difference analyses. Correlations between the two methods were as follows: the stereoacuity correlation coefficient was 0.779, and the stereopsis Phi coefficient was 0.624 (both *p* < 0.001). Autostereoscopic 3D technology demonstrated substantial consistency with the Titmus stereopsis test for both stereoacuity (κ = 0.659, *p* < 0.001) and stereopsis (κ = 0.619, *p* < 0.001). No significant differences were observed for stereoacuity (Z = −1.835, *p* = 0.066) and stereopsis (χ^2^ = 0.372, *p* = 0.542). Bland–Altman analysis revealed a mean bias of −25.60 arcsec (95% limits of agreement (LoA): −399.5 to 384.3 arcsec, Figure 3). Stereoacuity agreement was moderate for strabismus (κ = 0.553, *p* < 0.001), post-strabismus surgery (κ = 0.652, *p* < 0.001) and refractive error (κ = 0.638, *p* < 0.001). For stereopsis, κ = 0.589 (*p* < 0.001) in the strabismus group, κ = 0.684 (*p* < 0.001) in the post-strabismus surgery group, and κ = 0.578 (*p* = 0.012) in the refractive error group.

### 3.3. Comparative Statistical Results

**Figure 4:**
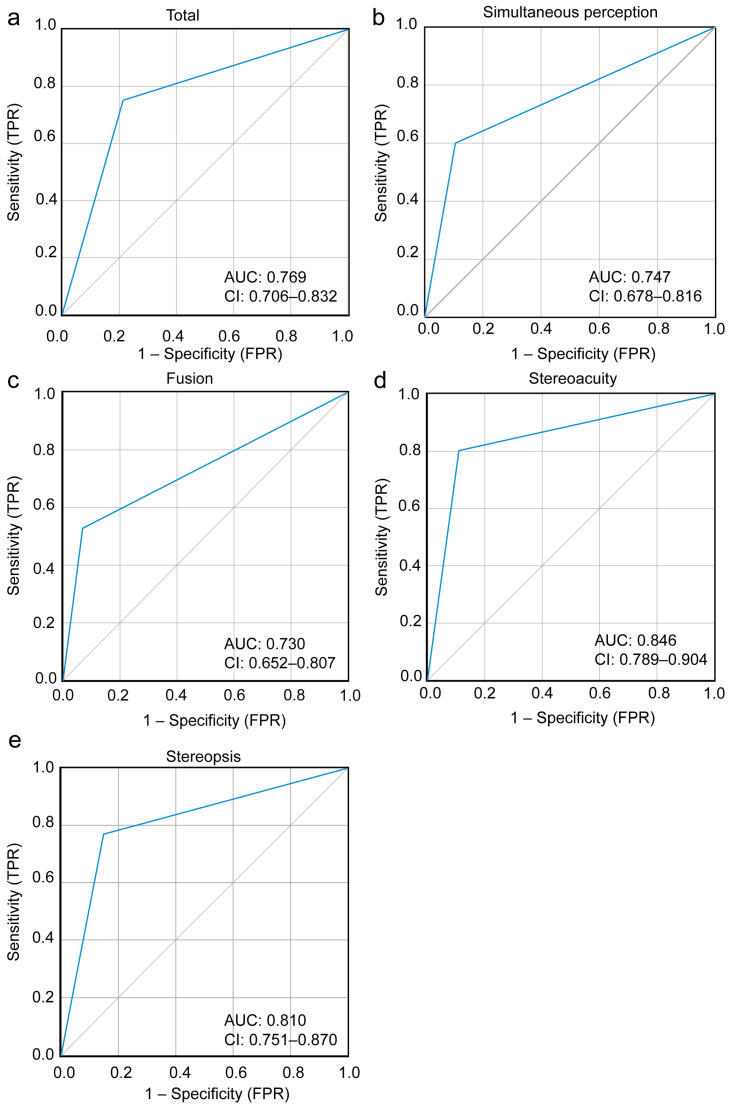
ROC curves for (**a**) overall binocular visual function, (**b**) simultaneous perception, (**c**) fusion, (**d**) stereoacuity and (**e**) stereopsis assessed by autostereoscopic 3D technology. (**a**) AUC = 0.769, 95% CI: 0.706–0.832. (**b**) AUC = 0.747, 95% CI: 0.678–0.816. (**c**) AUC = 0.730, 95% CI: 0.652–0.807. (**d**) AUC = 0.846, 95% CI: 0.789–0.904. (**e**) AUC = 0.810, 95% CI: 0.751–0.870. All *p* < 0.001. AUC, area under the curve; CI: confidence interval. The blue curve represents the ROC curve of the autostereoscopic 3D technology; the gray diagonal line represents the reference line of no discrimination (AUC = 0.50).

The positive predictive values (PPVs) for simultaneous perception, fusion, stereoacuity, and stereopsis assessed by autostereoscopic 3D technology were 78.3%, 77.6%, 80.3% and 80.6%, respectively. The corresponding negative predictive values (NPVs) were 77.5%, 81.4%, 88.2%, and 81.7%. The area under the curve (AUC) for detecting abnormal binocular visual function was 0.769 for overall binocular vision function, 0.747 for simultaneous perception, 0.730 for fusion, 0.846 for stereoacuity, and 0.810 for stereopsis (all *p* < 0.001, Figure 4). Simultaneous perception, fusion, stereoacuity, and stereopsis sensitivity were 60.0%, 52.8%, 81.7%, and 76.9%, respectively, with specificities of 77.9%, 93.1%, 89.0%, and 85.2% (Table 3).

## 4. Discussion

This study investigated the relevance and consistency of a novel autostereoscopic 3D technology in assessing binocular visual function, thereby evaluating the potential of autostereoscopic 3D technology for binocular visual function screening. Our findings indicated that this technology demonstrated significant positive correlations and moderate-to-high consistency with traditional anaglyphic 3D technology and the Titmus stereopsis test for simultaneous perception, fusion, stereoacuity and stereopsis. Furthermore, ROC analysis confirmed acceptable screening comparative performance for detecting binocular vision abnormalities.

Compared with anaglyphic 3D technology (red–green glasses), we observed strong positive correlations (φ > 0.5) between the two methods for both simultaneous perception and fusion. This confirmed the shared objective of these methods of evaluating higher-order binocular visual function. The consistency in simultaneous perception demonstrated that autostereoscopic 3D technology and anaglyphic 3D technology had equivalent capacity for separate eye visualization. The study by Chen et al. similarly showed that autostereoscopic 3D can achieve split-vision [27]. However, the significant difference in fusion assessment results (*p* = 0.001) warranted attention. Contrast and crosstalk of screening equipment affected fusion and stereopsis assessments [28]. Autostereoscopic 3D technology employed lenticular gratings to separate left and right eye images, providing stimuli closer to natural conditions. While horizontal parallax was precise, the absence of vertical depth cues may underestimate peripheral fusion function [29]. In contrast, red–green glasses maintained full-screen brightness and delivered stronger fusion stimuli, resulting in a higher fusion-positive rate (79% vs. 69%). This result is consistent with the McNemar test (*p* = 0.001).

Compared with the Titmus stereopsis test, autostereoscopic 3D technology showed higher concordance. No significant differences were observed for stereoacuity and stereopsis, with strong correlation (r = 0.779, φ = 0.624) and good agreement (κ > 0.6). These findings supported the reliability of autostereoscopic 3D technology as a stereopsis assessment tool [22]. The Bland–Altman analysis revealed a mean bias of only −25.6 arcsec between autostereoscopic 3D technology and the Titmus test, suggesting good population-level agreement. However, the 95% LoA had a wide range (±approximately 392 arcsec). This degree of variability exceeds the clinically significant difference threshold for stereoacuity and limits interchangeability at the individual patient level [22]. This suggests that while the two methods may yield comparable results at the population level, their current consistency for individual clinical decision-making remains limited. Several technical factors likely contribute to this variability. The Titmus test relies on polarized dissociation and contains monocular cues that may allow participants with limited stereopsis to respond correctly, potentially overestimating stereoacuity in some individuals. In contrast, the autostereoscopic system employs lenticular-grating separation without monocular cues, yielding a more stringent assessment of true binocular disparity processing. Moreover, the autostereoscopic system presents dynamic, continuously variable disparities under computerized protocols, whereas the Titmus test uses static, discrete stereoacuity levels. The fundamental difference in stimulus architecture may produce divergent thresholds in participants with borderline or unstable fusion. In addition, the study cohort included a high proportion of participants with impaired stereopsis (e.g., 64% classified as level 4, ≥400 arcsec), which may have led to an over-expansion of the LoA. These findings carry important practical implications. For population-level screening (such as school-based programs or community surveys), the strong correlation (r = 0.779) and substantial categorical agreement (κ = 0.659) support the use of autostereoscopic 3D technology to efficiently classify individuals into broad stereoacuity categories (adequate vs. impaired vs. absent). However, for individual-level clinical decision-making (such as preoperative assessment of stereopsis in strabismic patients), the wide LoA precludes direct interchangeability between the two methods. For example, a patient measured at 100 arcsec by one method could reasonably fall anywhere between 0 and 400 arcsec by the other method, spanning the range from excellent to severely impaired stereopsis. Consequently, the autostereoscopic 3D technology should not replace the Titmus stereopsis test when requiring precise individual stereopsis measurement, such as for monitoring subtle changes. Nevertheless, its significant correlation and good classification consistency indicated suitability for screening purposes—reliably categorizing individuals into “normal”, “impaired”, or “lost” stereopsis.

The demographic composition of the cohort merits consideration when interpreting the agreement and screening metrics. The predominance of children (84.0%, mean age 11.4 ± 8.7 years) reflects the target population for school-based screening applications, but also introduces age-related variability in task comprehension and cooperation. Stereoacuity is known to mature in early childhood and may be more labile in younger participants, potentially contributing to the wide limits of agreement observed between the autostereoscopic 3D system and the Titmus test. The presbyopia, reduced pupil size, and altered accommodative response might differentially affect autostereoscopic vision. The limited representation of adults and the exclusion of participants older than 60 years preclude conclusions about performance in aging populations. The male-to-female ratio was relatively balanced (125:107, 53.9% vs. 46.1%), and this study did not observe any a priori reason to expect differential device performance by gender. However, formal subgroup analysis by gender was not performed. Moreover, participants with systemic diseases were excluded. The impact of chronic diseases on autostereoscopic 3D assessment remains to be determined.

Subgroup analysis across strabismus, post-strabismus surgery, and refractive-error participants showed moderate consistency (all κ > 0.55, *p* < 0.05), indicating stable performance across varying visual function statuses [30]. Notably, the demographic composition of our cohort was 84.0% children, 59.1% strabismus patients, and only 8.2% participants with uncomplicated refractive error. This cohort limits the generalizability of our findings. This distribution reflected a hospital-based clinical population enriched for binocular dysfunction rather than a community screening cohort. Consequently, several caveats apply to the interpretation and extrapolation of our results. Substantial agreement (κ = 0.659 for stereoacuity) partly reflects the high proportion of clear-cut abnormalities (level 4, 64%). In a low-prevalence community sample with predominantly subtle or borderline deficits, agreement coefficients and screening metrics might differ. The 81.7% sensitivity for stereoacuity may be artificially elevated because severe abnormalities are easier to detect than subtle deficits. In a general pediatric population where binocular dysfunction is less prevalent and less severe, positive predictive values would likely decrease, and the false-positive rate might increase. These findings indicated that the glasses-free and highly automated nature of the autostereoscopic 3D technology system for school and community screening was only applicable to populations with a high prevalence of binocular vision disorders (e.g., specialty clinics or high-risk school populations). Effectiveness in low-prevalence community screening remains unproven and requires dedicated validation. However, this study aimed to provide preliminary validation of the system rather than a population-based screening evaluation. The postoperative cohort (*n* = 76) aimed to evaluate performance across varying degrees of functional recovery. Children constituted the predominant group (*n* = 195, 84.0%) to reflect the target population for school-based screening applications. The refractive error group (*n* = 19) was limited in size due to recruitment constraints, acknowledging its sample size was insufficient for subgroup analysis.

Additionally, under conditions that required no glasses, offered automation and one-click results, the PPV and NPV of autostereoscopic 3D technology both exceeded 77%, with AUC values ranging from 0.73 to 0.85, indicating acceptable screening comparative performance. Specifically, the AUC for stereoacuity and stereopsis were 0.846 and 0.810, respectively. The results were consistent with findings from Liu et al. [18]. This confirmed that autostereoscopic 3D technology was a reliable tool for stereopsis preliminary screening. Notably, the Titmus stereopsis test was affected by monocular cues and overestimates stereoacuity. Therefore, further study should use the gold standard for binocular visual function (such as synoptophore or random-dot-based tests) as the AUC reference standard to further validate this finding. Fusion sensitivity was only 52.8%, suggesting a tendency to miss fusion deficits. This result may stem from the fact that the Worth 4-dot test (anaglyphic 3D technology) was prone to false fusion and suppression artifacts due to its reliance on color dissociation (red–green glasses). The red–green glasses may allow for peripheral fusion even when central fusion was impaired, potentially overestimating fusion capability. Therefore, the Worth 4-dot test imposed relatively lower demands on fusion function. Participants with poor fusion function may still achieve fusion by actively suppressing color interference through brain mechanisms [31,32]. In contrast, autostereoscopic 3D technology used lenticular gratings that demand greater fusional stability and precision and may therefore detect earlier or subtler fusion dysfunction. Additionally, the autostereoscopic 3D technology system with pupil tracking may experience image crosstalk between the left and right eyes due to head tilting during testing among some younger children. Future solutions will incorporate AI-based head position detection and utilize more suitable headrests and chin rests to stabilize head positioning. Conversely, the high specificity (93.1%) implies a low false-positive rate. Notably, our study employed a fixed testing sequence, which introduces potential sources of bias. By testing the autostereoscopic 3D technology system first—when participants were unfamiliar with stereoscopic tasks—we likely underestimated its true capability relative to reference standards that benefited from practice effects. Learning effects could have improved performance on subsequent anaglyphic and Titmus tests, while cumulative visual fatigue from prolonged near-work might have degraded later performance. The superior stereoacuity sensitivity (81.7%) and AUC (0.846) may partially reflect the benefit of testing when participants were fresh. The modest fusion sensitivity (52.8%) might partly reflect reference-standard inflation via practice or, alternatively, fatigue-induced degradation. Therefore, randomized testing sequences in future validation studies is needed to further indicate the consistency and screening performance of this system.

Furthermore, the autostereoscopic 3D technology system eliminates consumable costs (e.g., disposable glasses, printed test cards) and reduced personnel requirements may yield cost advantages at scale. A formal cost-effectiveness analysis comparing per-screen costs against conventional methods is needed to inform public health decision-making. The automated eye-tracking and standardized presentation protocols reduce reliance on highly trained orthoptists or ophthalmologists. This examiner independence is critical for scalability in resource-limited settings. Moreover, the glasses-free, hygienic design addresses a key barrier to school deployment, making campus screening potentially feasible.

There were several limitations of the present study. First, the overall sample size, while adequate for preliminary evaluation, was relatively small for some subgroup analyses (particularly the refractive error group with *n* = 19). Second, the fixed testing sequence may have introduced order effects. Learning/practice gains could have inflated performance on later reference tests, while visual fatigue could have degraded them. Learning/fatigue effects may have biased the comparative performance metrics. Third, longitudinal follow-up was not performed, preventing assessment of intra-individual consistency over time. In addition, the absence of cycloplegic refraction may have resulted in incomplete accommodation control, particularly in younger children. Future studies should: (1) employ randomized or counterbalanced testing sequences to eliminate order effects; (2) include larger, population-representative samples; (3) incorporate gold-standard reference methods (synoptophore, TNO test); (4) evaluate test–retest reliability; and (5) assess visual fatigue and user experience quantitatively.

In summary, this study confirms that autostereoscopic 3D technology is a reliable and effective tool for preliminarily assessing binocular visual function. It demonstrates moderate consistency with traditional methods and acceptable screening efficacy, while offering the advantages of being glasses-free and automated. Moreover, in populations with varying visual function statuses, the autostereoscopic 3D technology maintains good consistency in evaluating stereoacuity and stereopsis. Despite individual variability and differences from the anaglyphic 3D technology in fusion, these findings establish a solid foundation for integrating autostereoscopic 3D technology into clinical practice and screening programs, highlighting its broad potential in future ophthalmic diagnostics and visual-health monitoring.

## 5. Conclusions

This study provides a comprehensive validation of an all-in-one, glasses-free autostereoscopic 3D system for multi-domain binocular visual function assessment. The technology demonstrated significant correlation and moderate agreement with traditional methods in assessing binocular visual function, particularly for stereopsis assessment. However, the modest sensitivity for fusion and simultaneous perception indicate that this technology should currently be regarded as preliminary screening tool rather than a definitive diagnostic instrument. The autostereoscopic 3D technology system offers advantages for large-scale deployment, but further optimization and validation in representative community samples against gold-standard methods with lower disease prevalence are required before implementation in community or school screening programs. Specifically, future studies should: (1) employ randomized or counterbalanced testing sequences to eliminate order effects; (2) validate the system in larger, population-representative community and school-based cohorts with lower disease prevalence; (3) benchmark performance against gold-standard reference methods (e.g., synoptophore, TNO random-dot stereotest); (4) assess test–retest reliability and quantitatively evaluate visual fatigue and user experience across age groups; and (5) conduct formal cost-effectiveness analyses to inform public health decision-making.

## Figures and Tables

**Table 1:** Demographics and baseline characteristics of participants (*n* = 232).

Variable	Classification	Total
Age, y		11.4 ± 8.7 (3~60)
Gender	Male	125 (53.88%)
	Female	107 (46.12%)
SE	Right eye	−0.72 ± 2.45
	Left eye	−0.75 ± 2.71
Spherical power, D	Right eye	−0.35 ± 2.41
	Left eye	−0.35 ± 2.69
Cylindrical power, D	Right eye	−0.66 ± 0.81 D
	Left eye	−0.75± 0.84 D

y, years; D, diopter; SE, spherical equivalent; mean ± standard deviation.

**Table 2:** Agreement analyses between methods.

	Kappa	95% CI	Z-Value/χ^2^-Value	*p*-Value
Simultaneous perception	0.425 *	0.324–0.527	−1.863 †	0.062
Fusion	0.503 *	0.381–0.626	10.756 ‡	0.001
Stereoacuity	0.659 *	0.565–0.753	−1.835 †	0.066
Stereopsis	0.619 *	0.517–0.721	0.372 ‡	0.542

CI, confidence interval; χ^2^, Chi-Square; * Cohen’s Kappa test; † Wilcoxon’s signed-ranks test; ‡ McNemar test.

**Table 3:** Screening comparative performance metrics.

	PPV (%)	NPV (%)	AUC (95% CI)	Sensitivity (%)	Specificity (%)
Overall binocular visual function	/	/	0.769 (0.706–0.832)	/	/
Simultaneous perception	78.3%	77.5%	0.747 (0.678–0.816)	60.0%	77.9%
Fusion	77.6%	81.4%	0.730 (0.652–0.807)	52.8%	93.1%
Stereoacuity	80.3%	88.2%	0.846 (0.789–0.904)	81.7%	89.0%
Stereopsis	80.6%	81.7%	0.810 (0.751–0.870)	76.9%	85.2%

PPV, positive predictive values; NPV, negative predictive values; AUC, area under the curve; CI, confidence interval.

## Data Availability

The data that relates to this study are available from the corresponding author upon reasonable request.

## References

[1] Benhaim-Sitbon L., Lev M., Polat U. (2022). Binocular fusion disorders impair basic visual processing. Sci. Rep..

[2] Candy T.R., Cormack L.K. (2022). Recent understanding of binocular vision in the natural environment with clinical implications. Prog. Retin. Eye Res..

[3] Tang L., Li L., Li C., Yu Y., Shu N., Zhang L. (2025). Comparison of binocular visual function among patients with different types of anisometropia. Adv. Ophthalmol. Pract. Res..

[4] Cao Y., Ye J., Nie A., Sun D., Yang M.-m. (2025). Correlation between binocular vision function and visual fatigue in school-age children with myopic anisometropia. Sci. Rep..

[5] Hu B., Liu Z., Zhao J., Zeng L., Hao G., Shui D., Mao K. (2022). The Global Prevalence of Amblyopia in Children: A Systematic Review and Meta-Analysis. Front. Pediatr..

[6] Wang Y., Zhao A., Zhang X., Huang D., Zhu H., Sun Q., Yu J., Chen J., Zhao X., Li R. (2021). Prevalence of strabismus among preschool children in eastern China and comparison at a 5-year interval: A population-based cross-sectional study. BMJ Open.

[7] Evans B.J.W., Pentland L., Evans B.E.W., Edgar D.F., Shah R., Conway M.L. (2026). Binocular Vision Anomalies in Scotland at Age 3.5-5.5 Years: An Epidemiological Study. Ophthalmic Physiol. Opt..

[8] Elamurugan V., Shankaralingappa P., Aarthy G., Kasturi N., Babu R.K. (2022). Assessment of stereopsis in pediatric and adolescent spectacle-corrected refractive error—A cross-sectional study. Indian J. Ophthalmol..

[9] Xie R., Zhao F., Yu J., Luo B., Jiang Z., Qiu X., Cao Y., Yang Y., Chen K., Zhang Y. (2024). Naked-Eye 3-Dimensional Vision Training for Myopia Control: A Randomized Clinical Trial. JAMA Pediatr..

[10] Ciner E.B., Kulp M.T., Pistilli M., Ying G.S., Maguire M., Candy T.R., Moore B., Quinn G. (2021). Associations between visual function and magnitude of refractive error for emmetropic to moderately hyperopic 4- and 5-year-old children in the Vision in Preschoolers—Hyperopia in Preschoolers Study. Ophthalmic Physiol. Opt..

[11] Joo H.J., Choi J.J., Ro J.W., Choi D.G. (2022). Comparison of sensory outcomes in patients with successful motor outcome versus recurrent exotropia after surgery for intermittent exotropia. Sci. Rep..

[12] Csizek Z., Mikó-Baráth E., Budai A., Frigyik A.B., Pusztai Á., Nemes V.A., Závori L., Fülöp D., Czigler A., Szabó-Guth K. (2023). Artificial intelligence-based screening for amblyopia and its risk factors: Comparison with four classic stereovision tests. Front. Med..

[13] Hong H., Jang J., Lee D., Lim M., Shin H. (2010). Analysis of angular dependence of 3-D technology using polarized eyeglasses. J. Soc. Inf. Disp..

[14] Wu H., Jin H., Sun Y., Wang Y., Ge M., Chen Y., Chi Y. (2016). Evaluating stereoacuity with 3D shutter glasses technology. BMC Ophthalmol..

[15] Vancleef K., Read J.C.A., Herbert W., Goodship N., Woodhouse M., Serrano-Pedraza I. (2017). Overestimation of stereo thresholds by the TNO stereotest is not due to global stereopsis. Ophthalmic Physiol. Opt..

[16] Murata N., Toda H., Ubukata H., Takagi M., Tanaka C., Machinaga A., Miyajima M., Tatara S. (2024). Development of Automated Visual Acuity Measurement Using a Calibration-Free Eye-Tracking System. Cureus.

[17] Gramatikov B.I. (2017). Detecting central fixation by means of artificial neural networks in a pediatric vision screener using retinal birefringence scanning. Biomed. Eng. OnLine.

[18] Liu F., Zhao J., Han T., Shen Y., Li M., Liu J., Yang D., Fang Y., Yan L., Zhou X. (2021). Screening for Stereopsis Using an Eye-Tracking Glasses-Free Display in Adults: A Pilot Study. Front. Med..

[19] Vancleef K., Serrano-Pedraza I., Sharp C., Slack G., Black C., Casanova T., Hugill J., Rafiq S., Burridge J., Puyat V. (2019). ASTEROID: A New Clinical Stereotest on an Autostereo 3D Tablet. Transl. Vis. Sci. Technol..

[20] Koon O.H., Badarudin N.E., Chu B.-S. (2026). Application of Eye-Tracking Technology in Assessing Binocular Vision Function in Paediatric Populations: A Scoping Review. J. Eye Mov. Res..

[21] Chen W., Li R., Yu Q., Xu A., Feng Y., Wang R., Zhao L., Lin Z., Yang Y., Lin D. (2023). Early detection of visual impairment in young children using a smartphone-based deep learning system. Nat. Med..

[22] Wang Y., Zhong J., Cheng M., Li J., Ma K., Hu X., Li N., Liang H., Zhu Z., Zhou J. (2022). A novel clinical dynamic stereopsis assessment based on autostereoscopic display system. Ann. Transl. Med..

[23] Ramachandran V.S. (1976). Learning-like phenomena in stereopsis. Nature.

[24] Bak E., Yang H.K., Hwang J.M. (2017). Validity of the Worth 4 Dot Test in Patients with Red-Green Color Vision Defect. Optom. Vis. Sci..

[25] Arnoldi K., Frenkel A. (2014). Modification of the Titmus Fly Test to Improve Accuracy. Am. Orthopt. J..

[26] Fawcett S.L., Birch E.E. (2003). Validity of the Titmus and Randot circles tasks in children with known binocular vision disorders. J. Am. Assoc. Pediatr. Ophthalmol. Strabismus.

[27] Chen F., Qiu C., Liu Z. (2022). Investigation of Autostereoscopic Displays Based on Various Display Technologies. Nanomaterials.

[28] Schor C., Heckmann T. (1989). Interocular differences in contrast and spatial frequency: Effects on stereopsis and fusion. Vis. Res..

[29] Zhao W.X., Wang Q.H., Wang A.H., Li D.H. (2010). Autostereoscopic display based on two-layer lenticular lenses. Opt. Lett..

[30] Yang Y., Wu H. (2019). Screening for Stereopsis of Children Using an Autostereoscopic Smartphone. J. Ophthalmol..

[31] Kovács G., Raabe M., Greenlee M.W. (2008). Neural correlates of visually induced self-motion illusion in depth. Cereb. Cortex.

[32] Economides J.R., Adams D.L., Horton J.C. (2012). Perception via the deviated eye in strabismus. J. Neurosci..

